# Identification of the *NLP* Gene Family in *Populus euphratica* and Its Expression Analysis Under Drought Stress

**DOI:** 10.3390/ijms27073071

**Published:** 2026-03-27

**Authors:** Xinyue Long, Chen Qiu, Jianhao Sun, Tongrui Song, Jing Li, Hongyan Jin, Donghui Miao, Xiaoli Han, Zhijun Li, Zhongshuai Gai

**Affiliations:** 1 College of Life Sciences and Technology, Tarim University, Alar 843300, China; lxinyue051600@163.com (X.L.); qiuchentea@163.com (C.Q.); sunjianhaotea@163.com (J.S.); songtr20001209@163.com (T.S.); jing926819729@163.com (J.L.); j2601803054@163.com (H.J.); mdh1216g@163.com (D.M.); lilyan0509@163.com (X.H.); 2 State Key Laboratory Incubation Base for Conservation and Utilization of Bio-Resource in Tarim Basin, Alar 843300, China; 3 Desert Poplar Research Center of Tarim University, Alar 843300, China

**Keywords:** *Populus euphratica*, *NLP* gene family, drought stress

## Abstract

NIN-like protein (NLP) transcription factors are key regulators of plant nitrate signaling and stress responses. Although extensively studied in *Arabidopsis thaliana* and various crops, it has rarely been reported in woody plants, particularly in drought-tolerant tree species. In this study, 10 *PeNLP* genes were identified in the drought-tolerant tree *Populus euphratica* Oliv. through comparative genomics. These genes were unevenly distributed across seven chromosomes, and the gene-family expansion was mainly driven by whole-genome duplication (WGD). Analysis of conserved domains showed that PeNLPs contained 4–10 characteristic motifs, and most members possessed the typical RWP-RK and PB1-related domains. Collinearity analysis identified 18 *NLP* orthologous gene pairs between *P. euphratica* and its relatives (*Populus pruinosa* and *Salix sinopurpurea*), which exceeded the 15 pairs detected between *P. euphratica* and *A. thaliana*, indicating that the NLP family is more conserved within the Salicaceae. Phylogenetic analysis divided *PeNLPs* into three subfamilies, and their promoter regions harbored diverse *cis*-acting elements associated with hormone signaling, environmental stress, growth, and light response. Transcriptome and qRT-PCR analyses further demonstrated that *PeNLPs* were generally downregulated under drought stress. Overall, this study systematically characterized the evolution, structure, and drought responsiveness of the *PeNLPs*, providing a theoretical basis and genetic resources for improving nitrogen use efficiency and drought resistance in trees.

## 1. Introduction

NIN-like protein (NLP) transcription factors are fundamental regulators of plant nitrate signaling. They typically contain two conserved domains, the RWP-RK domain and the C-terminal PB1 domain. The RWP-RK domain functions as a DNA-binding domain that recognizes nitrate-responsive cis-elements, whereas the PB1 domain mediates protein–protein interactions through dimerization with other PB1-containing proteins [1,2,3,4]. Genome-wide analyses have revealed the presence of *NLP* gene families across a wide range of plant species, with varying numbers reported in model organisms and crops such as *Arabidopsis thaliana*, rice, maize, and tea plants [5,6,7,8,9,10]. These studies have collectively confirmed the essential roles of NLP transcription factors in adapting to varying nitrogen conditions [11,12].

Recent studies have suggested that *NLP* functions extend beyond nitrate signaling to abiotic stress responses, particularly drought stress [13]. For example, in wheat, drought-induced abscisic acid (ABA) signaling activates the kinase TaSnRK2.10, which phosphorylates *TaNLP7* and reduces its stability. Upon rewatering, particularly under nitrogen supplementation, TaSnRK2.10 is inhibited, thereby restoring *TaNLP7* function and promoting post-drought recovery [14]. In *Arabidopsis*, the *atnlp7* mutant exhibits enhanced water retention, smaller stomatal apertures, increased sensitivity to ABA, and a higher survival rate under drought conditions, suggesting that *AtNLP7* negatively regulates drought tolerance [15]. In addition, *AtNLP8* has been implicated in drought response. *AtNLP8* negatively regulates drought resistance by transcriptionally activating *PUB23*, and the *AtNLP8* mutant exhibits enhanced drought tolerance [16]. These findings indicate that *NLPs* may serve as critical hubs for integrating nitrogen nutritional status with drought stress adaptation.

*Populus euphratica* Oliv., a keystone species native to the riparian forests of arid and semi-arid regions, is renowned for its extraordinary tolerance to drought and salinity, making it an ideal model for studying stress adaptation in woody plants [17,18]. Despite its ecological importance and unique adaptive traits, the NLP transcription factor family in *P. euphratica* remains poorly characterized. Key questions regarding its evolutionary history, expression dynamics under water deficit, and potential contribution to drought tolerance remain largely unresolved. To address this gap, a genome-wide identification of *NLP* family members in *P. euphratica* was conducted. Comprehensive bioinformatic analyses were performed to characterize their gene structures, conserved domains, phylogenetic relationships, and cis-regulatory promoter elements. Furthermore, transcriptomic data were integrated with qRT-PCR validation to elucidate their expression profiles under drought stress. This study aims to reveal the evolutionary characteristics and drought response mechanisms of the *PeNLP* family. Our findings will not only provide a foundational understanding of nitrogen and stress signaling integration in trees but also offer valuable candidate genes for future molecular breeding efforts aimed at enhancing stress tolerance in forestry.

## 2. Results

### 2.1. Identification, Chromosomal Distribution, and Physicochemical Properties of the PeNLP Family Members

**Figure 1:**
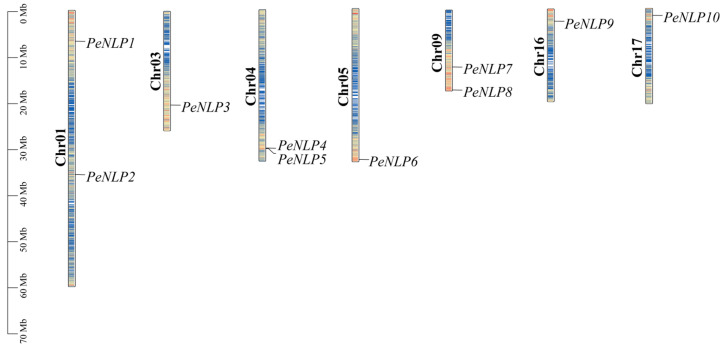
Chromosomal distribution of *PeNLP* genes.

**Figure 2:**
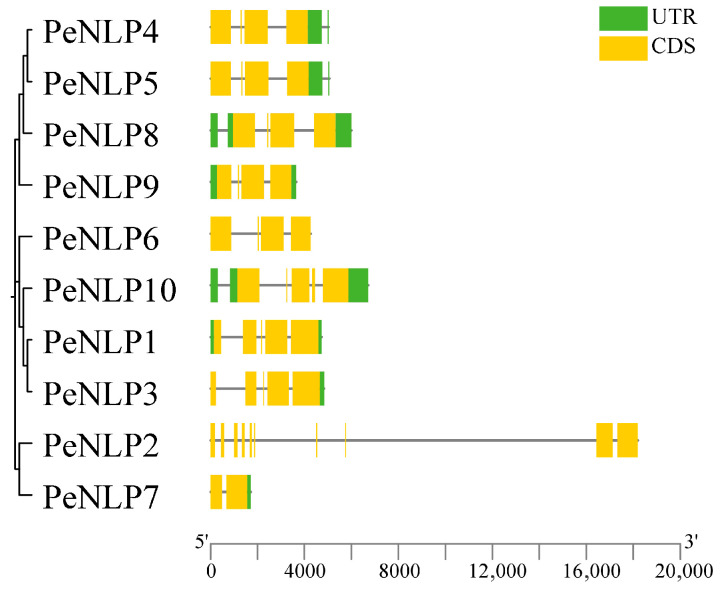
Exon–intron structure of *PeNLP* family.

In this study, a total of 10 *PeNLP* genes were identified in the *P. euphratica* genome. These genes unevenly distributed across seven chromosomes: chromosomes 1, 4, and 9 each contained two members, whereas the remaining chromosomes each harbored only one gene (Figure 1). Detailed information for each *PeNLP*, including sequence ID, genomic position, exon number, domain organization, and physicochemical properties, was provided in Table 1. Analysis of exon–intron structures showed that the number of exons ranged from 2 to 10 (Figure 2). Notably, *PeNLP2* contained a relatively longer intronic region compared with other members, which may reflect intron expansion events during its evolution. The protein lengths of PeNLPs ranged from 456 (PeNLP7) to 1007 (PeNLP1) amino acids, with corresponding molecular weights ranging from 51 kDa (PeNLP7) to 111 kDa (PeNLP1). The isoelectric points (pI) ranged from 5.77 (PeNLP2) to 7.90 (PeNLP9). All PeNLPs exhibited negative grand average of hydropathicity (GRAVY) values, indicating that they are hydrophilic proteins. Subcellular localization predictions indicated that most PeNLP proteins were localized in the nucleus, with the exception of PeNLP2, which was likely targeted to the chloroplast.

### 2.2. Duplication Type Analysis of PeNLPs

To analyze the evolution and expansion mechanisms of *PeNLPs*, their gene duplication types were investigated. The results showed that 8 *PeNLP* genes were derived from whole-genome duplication (WGD) or segmental duplication, whereas dispersed and tandem duplications accounted for only one gene each. These results suggested that WGD or segmental duplication played a major role in the expansion of these genes. This expansion may facilitate the adaptations of *P. euphratica* to arid environments.

### 2.3. Analysis of Protein Structure of the PeNLPs

**Figure 3:**
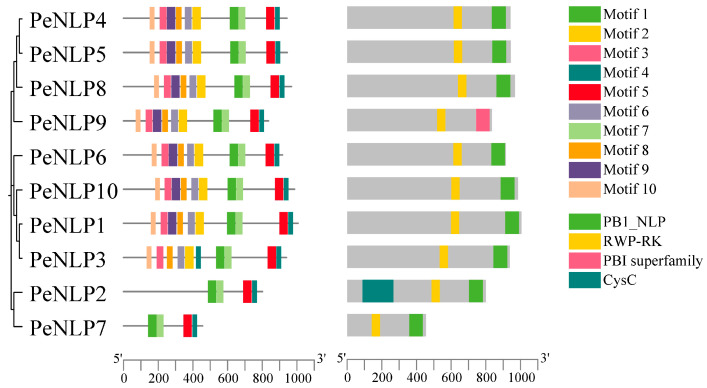
Conserved motif and domain architecture of the PeNLP protein family.

Analysis of conserved motifs revealed both commonality and diversity among the 10 PeNLP proteins (Figure 3). The number of identified motifs ranged from four to ten per protein, with Motifs 1, 4, 5, and 7 being conserved across all members, indicating their fundamental importance. Conversely, Motifs 2, 3, 6, 8, 9, and 10 were absent in some members, suggesting potential structural divergence during the evolution of the family. Pfam domain analysis confirmed that most PeNLPs contained the conserved RWP-RK domain and PB1_NLP domain. Notably, PeNLP2 additionally contained an additional CysC domain, while PeNLP9 differed from the other members by lacking the typical PB1_NLP domain and instead containing a PB1 superfamily domain.

### 2.4. Cis-Acting Element Prediction of PeNLPs

**Figure 4:**
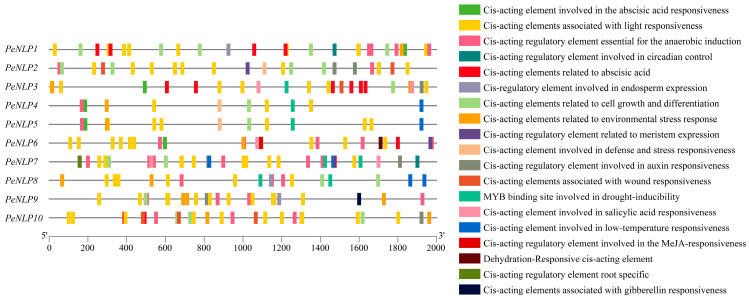
Predicted cis-regulatory elements identified in the 2 kb upstream promoter sequences of *PeNLP* family members.

Analysis of the 2000 bp upstream promoter sequences showed that cis-acting elements were mainly classified into four categories: hormone response, abiotic stress response, growth and development, and light response elements (Figure 4). The hormone-responsive elements primarily consisted of abscisic acid (ABA), salicylic acid (SA), methyl jasmonate (MeJA), auxin, and gibberellin responsive elements. Abiotic stress-related elements included MYB-binding sites involved in drought inducibility (MBS), defense and stress-responsive, low-temperature-responsive, and anaerobic induction-related elements. The growth and development-related elements involved cell growth and differentiation, meristem expression, and endosperm- and root-specific expression elements. Interestingly, MBS elements were detected only in a subset of *PeNLP* promoters rather than across all members, suggesting possible functional divergence in drought responsiveness within the *PeNLP* family. Additionally, light-responsive cis-elements were identified with high frequency across all *PeNLP* promoters. This finding suggested that light signals may participate in modulating the transcriptional activity of these genes.

### 2.5. Synteny Analysis of NLP Genes in P. euphratica and Related Species

**Figure 5:**
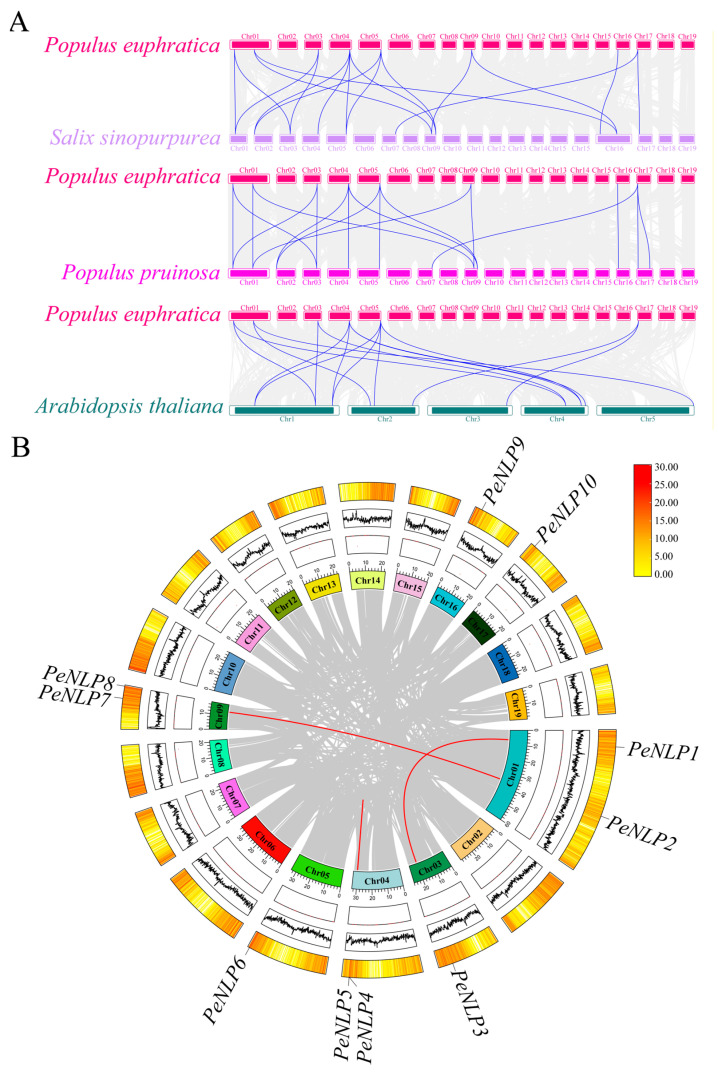
Syntenic analysis of *NLP* genes in *P. euphratica* and across multiple species. (**A**) Genome-wide synteny between *P. euphratica* and three representative species: *A. thaliana*, *P. pruinosa,* and *S. sinopurpurea*. Gray lines represent conserved syntenic blocks between genomes, while blue lines highlight orthologous *NLP* gene pairs; (**B**) Intraspecific synteny showing duplicated *NLP* gene pairs within the *P. euphratica* genome. Red lines indicate syntenic pairs of *PeNLP* fragment repeat genes.

To investigate the evolutionary dynamics of the *NLP* family, a total of 12 and 9 *NLP* genes were first identified in *Populus pruinosa* and *Salix sinopurpurea*, respectively. Synteny analysis identified 18 orthologous *NLP* gene pairs between *P. euphratica* and *P. pruinosa*. Similarly, 18 pairs were found between *P. euphratica* and *S. sinopurpurea*. In contrast, only 15 orthologous pairs were identified between *P. euphratica* and *A. thaliana* (Figure 5A). The syntenic relationships between *P. euphratica* and its closely related Salicaceae species were more significant, indicating high conservation of *PeNLPs* within the Salicaceae family. Intraspecific synteny analysis identified three pairs of paralogous genes: *PeNLP1*/*PeNLP3*, *PeNLP4*/*PeNLP5*, and *PeNLP2*/*PeNLP7*. This suggested that these gene pairs might have originated from duplication events and retained partial functional redundancy (Figure 5B). Furthermore, selection pressure analysis showed that the Ka/Ks ratios of all gene pairs were less than 1, indicating that the *NLP* gene family underwent strong purifying selection pressure during evolution (Table 2).

### 2.6. Phylogenetic Tree of PeNLPs

**Figure 6:**
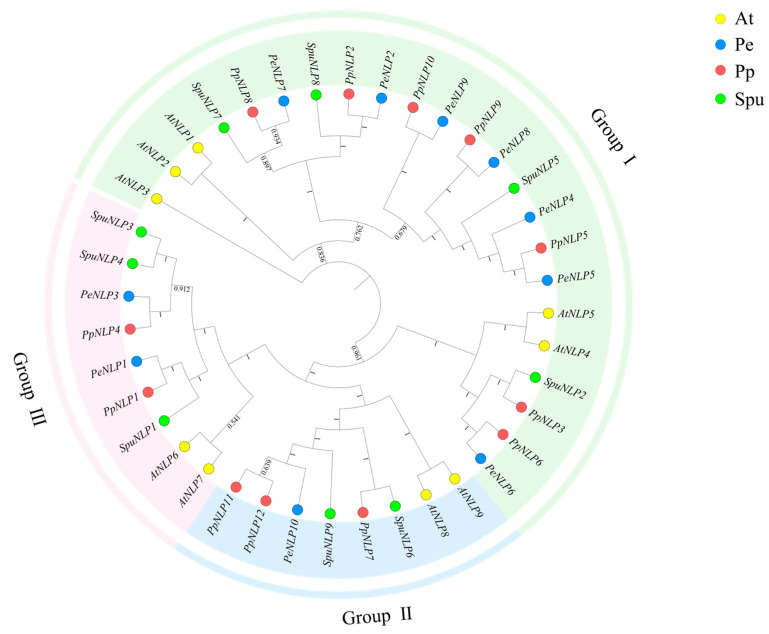
Phylogenetic tree depicting the evolutionary relationships of *NLP* family members across four representative species. Colored arcs mark major subgroup divisions. Circle colors correspond to species: At (*A. thaliana*), Pe (*P. euphratica*), Pp (*P. pruinosa*), Spu (*S. sinopurpurea*).

Phylogenetic analysis based on protein sequences classified PeNLP proteins into three subgroups (Figure 6). Seven members (PeNLP2, PeNLP4, PeNLP5, PeNLP6, PeNLP7, PeNLP8, and PeNLP9) belonged to Group I. PeNLP10 belonged to Group II, while PeNLP1 and PeNLP3 were assigned to Group III. PeNLP members were distributed across all three subgroups. The significantly higher number of members in Group I suggested that this subgroup might have undergone expansion during evolution. The phylogenetic tree showed that PeNLP proteins clustered more closely with PpNLP proteins.

### 2.7. Prediction of the Secondary Structure and Three-Dimensional Structure of the PeNLP Protein

**Figure 7:**
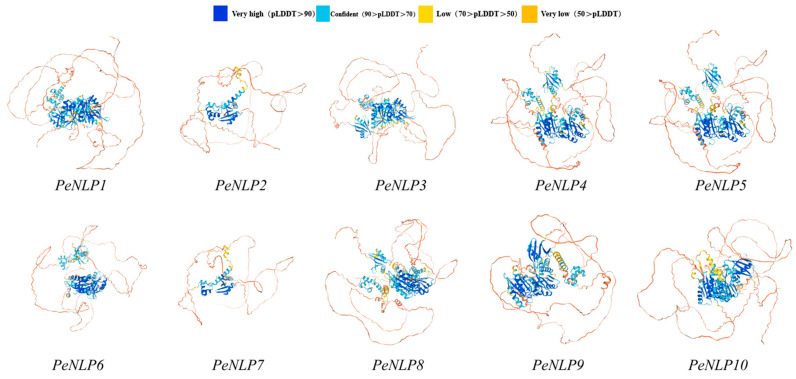
Protein 3D structure prediction model of *PeNLPs.* AlphaFold produces a per-residue confidence score (pLDDT) between 0 and 100.

Prediction of protein secondary structure revealed that all PeNLP members consist of four structural elements: α-helix, β-turn, random coil, and extended strand (Table 3). Among them, random coil (47.44–69.52%) accounted for the largest proportion, followed by α-helix (20.61–32.08%). In contrast, extended strand (7.89–15.48%) and β-turn (0.89–4.99%) were less abundant. The predominance of α-helices and random coils suggested that these structural components may play key roles in maintaining conformational stability and protein function. Three-dimensional (3D) structure prediction indicated that all PeNLP proteins possessed a similar overall conformation, characterized by a tightly folded core region and multiple external flexible segments. These results suggested that the spatial structures of PeNLP family were highly conserved (Figure 7).

### 2.8. Transcriptome Sequencing and Data Analysis of PeNLPs

**Figure 8:**
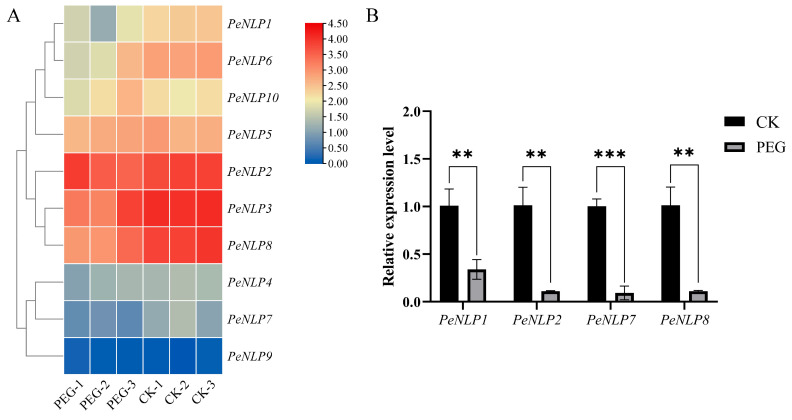
Expression patterns of *PeNLP* genes under PEG-induced drought stress in *P. euphratica*. (**A**) Heatmap showing the expression profiles of *PeNLP* family members at the seed stage under control (CK) and PEG6000 treatment. CK1-CK3 and PEG1-PEG3 represent three independent biological replicates of the control and PEG-treated samples, respectively. The color gradient from blue to red indicates relative expression levels (FPKM), with blue representing low expression and red representing high expression; (**B**) qRT-PCR analysis of selected *PeNLP* genes in two-month-old seedlings under PEG6000 treatment. The “***” indicates significant differences of *p* < 0.001; the “**” indicates significant differences of *p* < 0.01.

To investigate the transcriptional response of *PeNLPs* to drought, transcriptome data from a previously published study on *P. euphratica* at the seed stage under PEG6000 treatment were analyzed [19]. The results showed that the majority of *PeNLP* members were downregulated under drought stress, although the magnitude of downregulation varied among individual members (Figure 8A; Table S1). Specifically, *PeNLP2*, *PeNLP3*, and *PeNLP8* displayed high transcript abundance under control conditions but were significantly downregulated following stress treatment. Conversely, *PeNLP4*, *PeNLP7*, and *PeNLP9* showed low basal expression levels in the control group and either further declined or remained stably low under stress. Notably, *PeNLP1* also exhibited a significant decline. To further investigate the expression patterns at the seedling stage, representative *PeNLPs* were selected for qRT-PCR analysis in two-month-old seedlings. The expression of these selected genes showed a downward trend similar to that observed at the seed stage (Figure 8B). These findings suggested that PEG6000-induced drought stress generally suppresses the expression of *PeNLP* family members throughout the early development of *P. euphratica*.

### 2.9. Subcellular Localization of PeNLP1 in Nicotiana benthamiana

**Figure 9:**
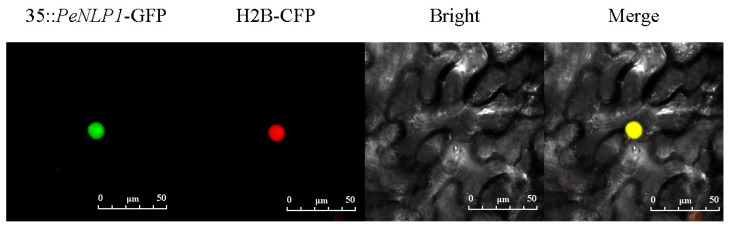
Nuclear localization of the 35S::*PeNLP1*-GFP protein in tobacco leaf epidermal cells. Green indicates fluorescent images of *PeNLP1* (35S::*PeNLP1*-GFP), red indicates nuclear localization signal (H2B-CFP), and yellow indicates the merged image (35S::*PeNLP1*-GFP/H2B-CFP).

Given that *PeNLP1* showed a significant transcriptional response to PEG6000 treatment and was considered a representative member of the *PeNLP* family for further functional characterization, a 35S::*PeNLP1*-GFP fusion construct was generated and transiently expressed in *N. benthamiana* leaves to determine its subcellular localization. Confocal laser scanning microscopy (CLSM) revealed that the GFP fluorescence signal was exclusively detected in the nucleus (Figure 9). These results indicated that *PeNLP1* was localized in the nucleus, consistent with its predicted role as a transcription factor.

## 3. Discussion

### 3.1. Evolution of the Salicaceae NLP Gene Family Was Predominantly Conserved, with Evidence of Subfunctionalization

Gene family expansion and differentiation represent critical evolutionary strategies for plants to adapt to complex environments [20,21,22]. In this study, 10 *NLP* members were identified in *P. euphratica*, of which 80% were originated from WGD or segmental duplication events. This finding was consistent with the evolutionary history of Salicaceae, which has undergone multiple polyploidization events [23,24]. All duplicated gene pairs exhibited Ka/Ks ratios below 1, indicating that the *NLP* gene family has been subjected to strong purifying selection during evolution. This evolutionary constraint likely ensures the structural integrity and retention of the RWP-RK and PB1 domains, thereby preserving the core roles of *PeNLPs* in nitrogen signaling [25,26]. Such structural and functional conservation has also been observed in other species, including rice and tea plant, where *NLP* members similarly maintain these hallmarks to function as nitrate signaling hubs [6,9]. However, within this framework of overall conservation, specific members such as *PeNLP2* exhibited distinct signs of subfunctionalization. In addition to its relatively longer intronic region, *PeNLP2* was uniquely predicted to localize to the chloroplast and to possess an additional CysC domain. The chloroplast is a pivotal site for nitrogen assimilation (nitrite reduction) and serves as a primary source of reactive oxygen species (ROS) [27,28]. It was therefore speculated that *PeNLP2* might perceive the redox state of the chloroplast via its CysC domain, thereby coupling photosynthesis and nitrogen metabolism with oxidative stress responses at the subcellular level [29]. In contrast, *PeNLP9* differed from the other members by lacking the typical PB1_NLP domain and instead containing a PB1 superfamily domain, further supporting the possibility of structural divergence within the family. These findings provided new insights into the subcellular specificity and functional divergence of the *NLP* family.

### 3.2. PeNLPs Downregulation May Represent a Potential “Throttling” Strategy for P. euphratica to Cope with Drought Stress

In this study, a consistent downward trend in the expression of most *PeNLP* genes was observed between the seed-stage transcriptome and the two-month-old seedling leaf qPCR data. Although these data sources represented distinct developmental stages and tissues, the shared response pattern suggested that the suppression of *PeNLPs* was a conserved reaction to drought stress throughout the early development of *P. euphratica*. This phenomenon echoes previous findings in *Arabidopsis*, where *AtNLP7* and *AtNLP8* were reported to negatively regulate drought resistance [15,16]. These results suggested that the suppression of *NLP*-mediated nitrogen signaling pathways under drought may serve as a conserved mechanism for stress response in angiosperms. Drought stress was known to induce stomatal closure and reduce photosynthetic rates [30], thereby reducing the immediate demand for nitrogen, particularly for nitrogen allocated to photosynthetic enzymes [31]. Under such conditions, maintaining high levels of nitrogen uptake and assimilation would not only consumed excessive energy and reducing power but also potentially exacerbated stress damage due to the accumulation of toxic nitrogen, such as ammonium toxicity. Therefore, under drought stress, *P. euphratica* may have suppressed *PeNLP* expression to limit nitrogen acquisition and assimilation, thereby facilitating a strategic shift from “growth-priority” to “survival-priority”. This “resource-saving” mechanism enabled the reallocation of limited resources toward direct stress-resistance processes, such as osmotic adjustment and reactive oxygen species (ROS) scavenging, thereby enhancing stress adaptability. The varying degrees of downregulation among *PeNLP* members may have reflected functional divergence or redundancy among different subfamilies in nitrogen signaling regulation. Overall, the suppression of *PeNLP* expression represented a resource trade-off strategy that *P. euphratica* had evolved during long-term adaptation to arid environments, whereby a dynamic balance was established between nitrogen utilization and stress defense to support survival and fitness in desert ecosystems.

### 3.3. Promoter Features Suggested PeNLPs as Key Nodes Integrating Multiple Environmental Signals

Promoter cis-element analysis provided a crucial perspective for understanding the transcriptional regulation of *PeNLPs*. The promoter regions of *PeNLPs* were enriched with various hormone-responsive elements (such as ABA, MeJA, and SA) as well as abiotic stress-responsive elements (such as drought and low temperature). These findings suggested that the expression of *PeNLPs* was regulated by the synergistic or antagonistic crosstalk among multiple stress and hormone signals. Notably, light-responsive elements were extensively presented in the promoters of all members. *P. euphratica* inhabited environments characterized by intense solar radiation, where light signaling was closely associated with its drought and salt tolerance. Light signals may have regulated *PeNLP* expression via phytochromes or through the status of the photosynthetic electron transport chain, thereby linking light energy supply (photosynthesis) with nitrogen demand (assimilation) at the transcriptional level. In this study, the *NLP* family members in *P. euphratica* were systematically identified, and their expression patterns and potential functions under drought stress were preliminarily revealed. Future work may focus on genetic validation, post-translational modification analysis, target gene identification, and functional characterization of cis-elements to systematically elucidate the molecular mechanisms and regulatory networks by which this family integrates nitrogen signaling and drought adaptation. Such efforts will provide a solid theoretical and genetic resource foundation for the genetic improvement of stress resistance in forest trees.

## 4. Materials and Methods

### 4.1. Identification, Chromosomal Mapping, and Physicochemical Analysis of PeNLPs

To identify *NLP* family members in *P. euphratica*, a comprehensive homology-based search were performed using the published genome assembly [32], along with publicly available genomic data from two related Salicaceae species: *P. pruinosa* (NCBI BioProject: PRJNA863418) and *S. sinopurpurea* [33,34]. Two complementary search strategies were employed. First, BLASTP (https://blast.ncbi.nlm.nih.gov/Blast.cgi?PROGRAM=blastp&PAGE_TYPE=BlastSearch&LINK_LOC=blasthome, accessed on 3 January 2026) searches (E-value < 1.0 × 10^−10^) were conducted against the three Salicaceae genomes using nine known *A. thaliana* NLP protein sequences as queries. Second, HMMER (http://pfam.xfam.org/search, accessed on 3 January 2026) searches were performed using the hidden Markov model profile of the *NLP* domain. Candidate sequences obtained from both approaches were further validated for the presence of complete and conserved NLP domains using Pfam batch search, NCBI Batch CD-Search, and the SMART online platform (http://smart.embl-heidelberg.de/, accessed on 6 January 2026). Only sequences containing both the characteristic RWP-RK and PB1 domains were retained as bona fide *NLP* family members.

Chromosomal locations, gene identifiers, and positional coordinates of the identified *PeNLP* genes were extracted from the genome annotation files using TBtools (v2.097). Gene density was also calculated, and the chromosomal distribution of *PeNLP* genes was visualized using the Gene Location Visualize function within TBtools.

The physicochemical properties of the deduced PeNLP proteins, including theoretical molecular weight (Mw) and isoelectric point (pI), were predicted using the ExPASy online server (https://www.expasy.org/). Subcellular localization was predicted using the WoLF PSORT web tool (https://wolfpsort.hgc.jp/, accessed on 6 January 2026).

### 4.2. Analysis of Gene Duplication Patterns

To investigate the expansion mechanisms of the *NLP* family in *P. euphratica*, WGD events were identified using MCScanX (https://megasoftware.net/, accessed on 10 January 2026). Duplication patterns for individual *PeNLP* genes were then classified into categories such as WGD/segmental, tandem, proximal, and dispersed duplication. The relative contributions were also statistically analyzed.

### 4.3. Analysis of Gene Structure and Conserved Motifs

Conserved motifs in PeNLP proteins were identified using the MEME online suite (http://meme-suite.org/, accessed on 11 January 2026), with the maximum number of motifs set to 10 and default parameters for all other settings. Gene structural organization was determined by comparing coding sequences with their corresponding genomic sequences. Motif distributions patterns and gene structures were visualized using TBtools to facilitate comparative analysis.

### 4.4. Promoter Cis-Element Analysis

To explore potential transcriptional regulation of *PeNLP* genes, 2000 bp upstream sequences from the translation start site were extracted for each gene. These putative promoter regions were submitted to the PlantCARE database for cis-acting regulatory element prediction. The types and abundances of identified elements were visualized using TBtools.

### 4.5. Synteny Analysis

Syntenic relationships between *P. euphratica* and three representative species (*P. pruinosa*, *S. sinopurpurea*, and *A. thaliana*) were analyzed to assess the evolutionary conservation of the *NLP* family. Homologous gene pairs were first identified using BLASTP searches (E-value < 10^−5^). Syntenic blocks containing *NLP* genes were then detected using TBtools, and the resulting inter- and intra-specific syntenic relationships were visualized to reveal orthologous and paralogous gene pairs.

### 4.6. Phylogenetic Analysis of the PeNLPs

For phylogenetic analysis, NLP protein sequences from *P. euphratica*, *P. pruinosa*, *S. sinopurpurea*, and *A. thaliana* were collected. Conserved *NLP* domains were extracted based on SMART database annotations. The extracted domain sequences were aligned using the Clustal algorithm implemented in MEGA-X (v11.0.13) software with default parameters. Based on the Neighbor-Joining (NJ) method with 1000 bootstrap replicates, a phylogenetic tree was constructed and subsequently visualized using the iTOL online platform.

### 4.7. Prediction of Protein Secondary and Tertiary Structures

Secondary structures of PeNLP proteins were predicted using the SOPMA web server, which provides percentages of α-helix, β-turn, random coil, and extended strand elements. Three-dimensional (3D) structural models were generated by homology modeling using the SWISS-MODEL server, enabling assessment of the conserved spatial conformation of the *PeNLP* family.

### 4.8. Transcriptome Analysis and qRT-PCR Validation

To investigate the expression patterns of *PeNLP* genes under drought stress, transcriptome data were obtained from a previously published study on *P. euphratica* seeds treated with PEG6000, with untreated seeds used as controls [19]. In that dataset, CK1-CK3 and PEG1-PEG3 represent three independent biological replicates of the control and PEG-treated groups, respectively. The expression profiles of *PeNLP* genes were extracted from this published dataset and used for heatmap analysis.

Two-month-old *P. euphratica* seedlings were selected for PEG 6000 treatment for five days to simulate drought stress, with a control group established. Each group included three biological replicates. After treatment, three leaves of similar size were collected, immediately frozen in liquid nitrogen, and stored at −80 °C until further analysis.

For qRT-PCR validation, total RNA was reverse-transcribed using the SPARKscript RT Plus Kit with gDNA Eraser (SparkJade, Jinan, China). Each 10 µL qPCR reaction contained 5 µL 2 × SYBR Green qPCR Mix (SparkJade, China), 0.2 µL each of forward and reverse primers, 3.1 µL ddH_2_O, and 1.5 µL cDNA template. Amplicon sizes ranged from 80 to 150 bp. Relative expression levels were calculated using the 2^−ΔΔCt^ method with *PeActin* as the internal reference gene [35]. The primer sequences used were listed in Table S2.

### 4.9. Subcellular Localization of PeNLP1

To experimentally validate the subcellular localization of *PeNLP1*, the coding sequence was cloned into a 35S::GFP fusion vector to generate the 35S::*PeNLP1*-GFP. The recombinant plasmid was transformed into Agrobacterium tumefaciens strain GV3101 using the freeze–thaw method. Agrobacterium cultures carrying the fusion construct and the nuclear marker H2B-CFP were separately grown in LB medium with appropriate antibiotics, then harvested by centrifugation and resuspended in infiltration buffer (10 mM MES, pH 5.6; 10 mM MgCl_2_·6H_2_O; 50 µM acetosyringone) to an OD_600_ of approximately 1.0. After incubation in the dark for 3 h to activate virulence genes, the two cultures were mixed in equal volumes and co-infiltrated into the abaxial side of 4-week-old *N. benthamiana* leaves using a needleless syringe. Infiltrated plants were kept in the dark for 12 h and then transferred to normal growth conditions (22 °C, 16 h light/8 h dark) for 36 h. GFP fluorescence was observed under a confocal laser scanning microscope with excitation at 488 nm and emission detection at 500–550 nm [19].

## 5. Conclusions

This study provided a systematic characterization of the *NLP* gene family in *P. euphratica*. The *PeNLP* family was found to be evolutionarily conserved within the Salicaceae, with WGD serving as the primary driver of its expansion, while also showing evidence of subfunctionalization. The predicted chloroplast localization of *PeNLP2* suggested potential functional specialization associated with adaptation to desert environments. The predominant downregulation of *PeNLPs* under drought stress across different developmental stages indicated that suppression of nitrate signaling may represent an important strategy for *P. euphratica* to balances growth and survival under drought conditions. Collectively, these findings provided a comprehensive foundation for understanding the molecular mechanisms underlying nitrogen signaling and drought adaptation in woody plants, and offered valuable candidate genes for future genetic improvement of stress tolerance in forestry.

## Figures and Tables

**Table 1:** Characteristics of *PeNLPs*.

Sequence ID	Gene Name	Genomic Position	Number of Exons	Main Domain	Number of Amino Acid	Molecular Weight	Theoretical pI	Instability Index	Aliphatic Index	GRAVY	Prediction of Subcellular Localization
PeuTF01G00847.1	*PeNLP1*	Chr01:	5	RWP-RK (599–646); PB1_NLP (912–992)	1007	111,020.65	5.81	49.58	76.5	−0.398	nucl
		6745911–6750641									
PeuTF01G02880.1	*PeNLP2*	Chr01:	10	RWP-RK (487–535); PB1_NLP (703–784)	801	89,104.16	5.77	48.1	73.4	−0.517	chlo
		35707921–35726111									
PeuTF03G01419.1	*PeNLP3*	Chr03:	5	RWP-RK (534–582); PB1_NLP (844–924)	939	103,654.92	5.82	52.28	72.59	−0.476	nucl
		20362334–20367178									
PeuTF04G01931.1	*PeNLP4*	Chr04:	4	RWP-RK (614–662); PB1_NLP (835–916)	943	104,929.99	6.7	49.58	71.68	−0.526	nucl
		30071926–30076958									
PeuTF04G01949.1	*PeNLP5*	Chr04:	4	RWP-RK (616–664); PB1_NLP (837–918)	945	104,996.04	6.39	51.5	72.95	−0.503	nucl
		30177102–30182164									
PeuTF05G02327.1	*PeNLP6*	Chr05:	4	RWP-RK (613–661); PB1_NLP (832–911)	917	101,214.26	5.89	57.13	75.25	−0.444	nucl
		32790743–32795009									
PeuTF09G00891.1	*PeNLP7*	Chr09:	2	RWP-RK (142–190); PB1_NLP (358–437)	456	51,267.26	5.82	59.77	70.53	−0.67	nucl
		12376778–12378500									
PeuTF09G01660.1	*PeNLP8*	Chr09:	4	RWP-RK (640–688); PB1_NLP (861–942)	969	107,473.98	6.22	50.03	70.03	−0.474	nucl
		17371570–17377576									
PeuTF16G00365.1	*PeNLP9*	Chr16:	4	RWP-RK (519–567);	836	93,488.91	7.9	43.49	75.45	−0.407	nucl
		2663580–2667234		PB1 superfamily (744–823)							
PeuTF17G00157.1	*PeNLP10*	Chr17:	5	RWP-RK (601–649); PB1_NLP (886–966)	987	108,316.75	5.54	49.22	72.34	−0.395	nucl
		1531472–1538192									

**Table 2:** Selection pressure analysis of *PeNLP* gene pairs.

Gene_1	Gene_2	Ka	Ks	Ka/Ks
*PeNLP1*	*PeNLP3*	0.094	0.273	0.345
*PeNLP4*	*PeNLP5*	0.011	0.017	0.658
*PeNLP2*	*PeNLP7*	0.115	0.237	0.485

Note: The abbreviations Ka (non-synonymous) and Ks (synonymous) are used to denote substitution rates.

**Table 3:** Predicted secondary structure composition of PeNLP proteins.

Gene Name	Percentage (%)				Distribution of Secondary Structure Elements by Amino Acid Position (aa)
	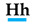	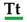	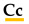	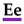	
*PeNLP1*	21.55	0.89	68.72	8.84	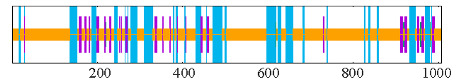
*PeNLP2*	32.08	4.99	47.44	15.48	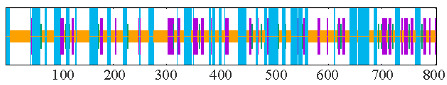
*PeNLP3*	23.64	2.34	65.92	8.09	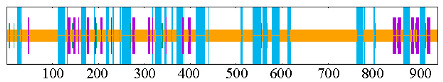
*PeNLP4*	25.56	1.38	63.52	9.54	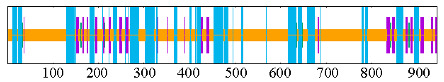
*PeNLP5*	22.01	1.06	68.25	8.68	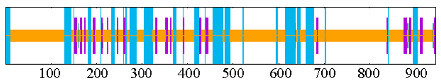
*PeNLP6*	25.52	1.85	64.34	8.29	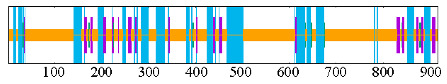
*PeNLP7*	20.61	1.97	69.52	7.89	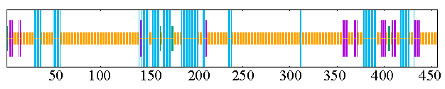
*PeNLP8*	24.56	1.34	64.91	9.18	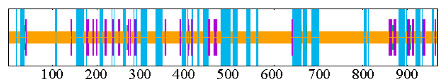
*PeNLP9*	25.72	1.32	63.52	9.45	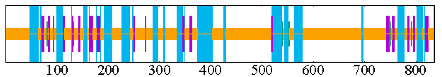
*PeNLP10*	24.52	1.01	66.16	8.31	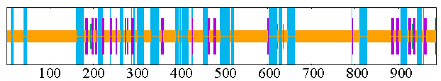

Note: Hh (blue, α-helix), Tt (green, β-turn), Cc (yellow, random coil), and Ee (purple, extended strand).

## Data Availability

The original contributions presented in this study are included in the article. Further inquiries can be directed to the corresponding authors.

## Supplementary Materials

The following supporting information can be downloaded at: https://www.mdpi.com/article/10.3390/ijms27073071/s1.
